# The Sixth Annual Report of the Registrar-General of Births, Deaths, and Marriages, in England, 1845

**Published:** 1846-01

**Authors:** 


BRITISH AND FOREIGN

### MEDICAL REVIEW,

FOR JANUARY, 1846.

### PART FIRST.

### Analytical and Critical Reviews.

### Art. I.

1. *The Sixth Annual Report of the Registrar-General of Births, Deaths, and Marriages, in England,* 1845.
2. *Contributions to Vital Statistics: being a Development of the Rate of Mortality and the Laws of Sickness; from original and extensive Data, procured from Friendly Societies, showing the Instability of Friendly Societies, "Odd Fellows," "Rechabites," &c., with an Inquiry into the Influence of Locality on Health.* By F. G. P. Neison, f.l.s., &c., Actuary to the Medical Invalid and General Life Office. *Read before the Statistical Society, March* 17, 1845.
3. *Facts connected with the Social and Sanitary Condition of the Working Classes in the City of Dublin, with Tables of Sickness, Medical Attendance, Deaths, Expectation of Life, &c. &c.; together with some Gleanings from the Census Returns of* 1841. By Thomas Willis, f.s.s. *Dublin,* 1845.
4. *On the Duration of Life among the Families of the Peerage and Baronetage of the United Kingdom.* By William A. Guy, m.b. Cantab., &c., &c. (*Extracted from the Journal of the Statistical Society for March* 1845.)

It is always an agreeable task to pass under review a periodical publication which bears on the face of it evidence of a continual striving after progress and improvement. This pleasure is provided for us year by year in the Reports of the Registrar-General. The one which is now before us is the largest, and, in many respects, the most interesting and important of the six which have issued from Somerset House. It contains, in addition to the usual Report and detailed abstracts of births, deaths, and marriages in England and Wales, a series of foreign official returns, of which the greater part was procured for the Registrar-General by the Earl of

Aberdeen (at the instance of Sir James Graham,) through the Foreign or British Ministers, and the remainder from Dr. Elliott of Stockholm, M. Hoffman of Berlin, and M. Moreau de Jonnes of Paris. Returns have been obtained from Sweden, Norway, Denmark, Russia, Austria, Prussia, Saxony, Hanover, Frankfort, Bavaria, Wurtemburg, Netherlands, Belgium, France, Portugal, and the United States of America. We shall presently have occasion to notice some of the results obtained from these sources.

After the abstracts for England and the several continental returns, which together occupy 500 closely-printed octavo pages, we are favored with a series of very valuable dissertations by Mr. Farr, headed respectively "Public Health," "Summary of results deduced from the English Life Table," "On the properties and applications of the New Life Table, and an account of some improvements in its form," "The *annual mortality* and the *mean age at death*," "English Joint-Life Table," "Life Insurance," &c. &c. These dissertations occupy upwards of 150 pages, and abound in tables, which must be of the first importance to the actuary.

These tables are not of a nature to admit of analysis in this place; we must, therefore, content ourselves with this reference to them, reserving only certain parts of the essays, that entitled "the annual mortality, and the mean age at death," among the number, for criticism in connexion with Mr. Neison's valuable "Contributions to Vital Statistics." We cannot, however, refrain from pointing, in passing, to the valuable table at p. 655 of the Report, which, for the first time, presents us with the mean age at death of the inhabitants of England living at every age, and contains other useful columns. Some interesting applications of this table will be found at p. 661 of the 8vo edition of the Report.

There were registered, in England and Wales, in the year 1842, 118,825 marriages, 517,739 births, and 349,519 deaths, being an excess of births over deaths of 168,220. Taking the average of four years, 1839-1842, 1 person in 130 was married, 1 in 45 died, and 1 child was born to 31 persons living. The marriages have decreased during that period from 1 in 126 to 1 in 136, the births have increased up to 1841, and the deaths were highest in 1840, and lowest in 1841. The years 1841 and 1842 have exhibited little difference in the relative number either of births or deaths: 1 in 64 of the male population, and 1 in 66 of the female population married annually; there was 1 birth to 15 males and to 16 females living; and the mortality of males was 1 in 44, and of females 1 in 47, or, to be more accurate, 2·294 and 2·124 per cent.

An interesting comparison is instituted between the marriages, births, and deaths in England and the four principal European states: *France, Prussia, Austria,* and *Russia*. It appears that during the last few years to which the returns refer, there have been fewer marriages in England than in any of the larger European states. While in England there is only 1 marriage in 131 persons living, there is 1 in 124 in Austria, 1 in 121 in France, 1 in 113 in Prussia, and 1 in 99 in Russia. The births, as might be anticipated, are also in defect in England. The relative numbers being as follows: England 1 birth in 35 persons living, Prussia 1 in 27, Austria 1 in 26, Russia 1 in 23, France alone presenting the equal ratio of 1 in 35, a conclusive proof that in France there are fewer children to a marriage than in England.

The number of illegitimate children registered in England and Wales in 1842 was 34,796; of these a somewhat larger proportion were males than in the case of legitimate births, for it appears that while among legitimate children there were 20 boys to 19 girls born alive, among illegitimate children there were 21 to 20. In all England there were 6·7 illegitimate births in 100 births, and it is not a little remarkable that nearly all the large towns stand below this average. The highest proportion anywhere observed is 18 per cent., the lowest somewhat above 1 per cent. The proportion of illegitimate children varies not less remarkably in different nations, for while Sardinia has so low a proportion as 2 in a 100, Bavaria has no less than 20 in the 100. Of 13 European states England takes the fourth place in regard to continence. The returns for the continental states confirm the well-known fact of the high mortality of illegitimate children. Thus, in Saxony, while 464 in 10,000 legitimate children were still-born, 616 in the same number of illegitimate children were still-births. The deaths during the first year in Saxony, Sweden, and Stockholm respectively, were 26, 16, and 26 per cent. of legitimate to 34, 27, and 40 illegitimate.

It now only remains to speak of the mortality of England, as compared with the continental states. The average annual mortality of the English population in the five years, 1838-42 was 2·209 per cent., or nearly 1 in 45; but in 1842 it was 2·167, or nearly 1 in 46. This is a more favorable rate of mortality than that which prevails in the chief European states. In France it is 1 in 42, in Prussia 1 in 38, in Austria 1 in 33, and in Russia 1 in 28.

The result of the registration for 1842, then, as compared with former years, and the movement of the English population as compared with that of the principal states of Europe, appears on the whole to be satisfactory. It is clear that we possess advantages which it is our duty and our interest to improve to the utmost. It will not do to compare ourselves only with our neighbours; we must take a much higher standard than that which any continental state affords, and be satisfied for the whole country with nothing short of the rate of mortality of its most favoured divisions. In the South-western and South-eastern divisions the mortality is stated at 1 in 52, and in no less than four counties (Sussex, Dorsetshire, Devonshire, and North Wales,) it is as low as 1 in 54. The time will, we trust, arrive when the entire country shall boast as low a mortality. In the mean while let it be our standard of excellence.

We have still to notice a valuable return of violent deaths and suicides registered in 1840. They amount to no less than 10,881, of which 900 were cases of suicide, and 65 were murders. If we take the instrument or means of death employed by suicides, the following will be the order of their frequency: hanging, strangling, and suffocation, 381; poisons 161; wounds 129; drowning 107; gun-shot wounds 45; leaps from heights 18; unascertained 60. Of the cases of suicides by poison 26 were by arsenic, 19 by opium, 3 by oxalic acid, and 113 by other poisons. Among the accidental deaths the following are deserving of notice: overdose of opium or laudanum, given or taken by mistake, 39; oil of vitriol, given or taken by mistake, 15; drinking boiling water (children,) 23; making in all 77 accidental deaths from these three causes.

Having now given our usual abstract of the Registrar-General's Report, as far as it refers to the number of births, deaths, and marriages, we proceed to present our readers with some account of the other works which stand at the head of the present article, especially of the very valuable and highly interesting publication of Mr. Neison.

We have already introduced Mr. Neison to our readers in an article on the "Vital Statistics of England and Wales;"* and we then took occasion to express our dissent from the extreme views which he had adopted. We regret that he has imposed the same duty upon us on the present occasion; but ere we enter upon the discharge of it, we must express the strong sense we entertain of the value of Mr. Neison's labours. His facts and calculations may be implicitly relied on, though his conclusions are sometimes open to objection; and his liberality in stimulating and rewarding his contributors by prizes from his own purse must not pass without notice. The "contributions" too, consisting, to so great an extent, of tables, must have been published at great cost, and with little hope of a pecuniary return; so that on the whole, Mr. Neison has placed all who may profit by his labours under many and great obligations. The members of friendly societies, "Odd Fellows," "Rechabites," &c. must own themselves, in a peculiar manner, his debtors; and they will do well to listen to Mr. Neison's warning, ere it be too late.

The "contributions" consist of two parts, of which the first treats of the duration of life among different classes, and the second of the prevalence of sickness among the members of benefit societies.

The first part comprises several life-tables, which Mr. Neison uses as the foundations on which to build his deductions. By throwing his facts into this form he has obtained by far the most satisfactory data, and has avoided the error which he himself pointed out in an essay formerly referred to, of instituting comparisons from which an essential element (the ages of the living,) was omitted. On the superiority of life-tables to all other data for determining the sanitary state of nations, or classes of persons, Mr. Farr makes some very just observations. In the chapter of the 'Report,' headed "the annual mortality" and "the mean age at death," he proves satisfactorily that the mean age of the living has always been esteemed by the highest authorities as a necessary element in every just comparison of class with class and country with country; and he demonstrates the necessity of taking this circumstance into account, by comparing the expectation of life, as calculated from the ages both of the living and the dying, with the average age at death, and the number of living out of which one death takes place annually. From this comparison it results that there is a much nearer correspondence between the expectation of life and the mortality, than between the expectation of life and the average age at death. Thus, if we compare England, France, and Sweden, we find that they hold the same relation to each other in regard both to the expectation of life and the mortality, the order being England, France, Sweden; but in respect of the average age at death the three countries occupy a different position: France having the highest average age at death, Sweden holding the second rank, while England stands at the bottom of the scale. So also if we compare England, Surrey, the Metro-

* British and Foreign Medical Review, No. XXXV, p. 198.

polis, and Liverpool, there is the same correspondence between the expectation of life, as shown by the life-tables, and the mortality, while the mean age at death in England and the Metropolis is precisely the same, viz., 29 years, though the expectation of life for the former is 41 years, and for the latter only 37 years.

The necessity of employing life-tables as our tests of the sanitary condition of different classes, and of the inhabitants of different localities, is further illustrated by the anomalous results obtained by employing only the mean age at death. According to this latter method, Greenwich is the healthiest district of the Metropolis, for the obvious reason, that there is an accumulation of old men at Greenwich Hospital, who, dying at advanced ages, make the mean age at death high. By using the same one-sided test, Rotherhithe is made to appear more healthy than Islington, Marylebone, and Pancras; and Whitechapel takes a more favorable sanitary position than St. James's district and the city of London.

In adopting the life-table, therefore, as his test of the sanitary condition of the classes which he makes the subjects of comparison, Mr. Neison has made choice of the only perfectly unobjectionable standard; and we shall willingly admit that his facts and tests are unexceptionable, though, as we have already hinted, we shall not be quite so easily satisfied with his arguments.

Mr. Neison first provides himself with a standard of comparison in the shape of a life-table of England and Wales, founded on the census for 1841, compared with the deaths as given in the 2d, 3d, 4th, and 5th Reports of the Registrar-General. Our readers may recollect that Mr. Farr's life-table was calculated on the mortality of the year 1841 alone; it is evident, therefore, that the average employed by Mr. Neison must form "a broader and more satisfactory basis on which to found a measure of the duration of life in this country." On comparing the two life-tables with each other, Mr. Neison's table will be found to give a higher expectation, both for males and females, throughout the whole of life. At 60 years of age the difference amounts for the male to one year, and for the female to somewhat more than a year. It is somewhat singular that the difference between Mr. Neison's table and the Carlisle table, is, taking one period of life with another, still less than that which exists between the two tables just compared.

A standard of comparison thus obtained, our author proceeds to develope his views, and to reveal his scepticism with regard to the "supposed influence of locality on the duration of life;" and he justly observes that hitherto no public means have been employed to solve this question.

"For the progress of vital statistics it unfortunately happens, that the public records of this country are kept with very little regard to method or unity of plan. The Report of the Census may certainly in itself be regarded as a very complete document; and perhaps no other country possesses such excellent mortuary registers; yet for almost every purpose of exact calculation, both documents are nearly useless. No two things should have been more intimately related in design and classification, than the census of the people and the registration of deaths. Still they seem to have been compiled without any regard to each other. For example, if it were required to compare any two counties in England—a manufacturing with an agricultural county—an inland with a coasting county—in order to determine the relative value of life in the respective populations, it cannot at the present time be done. The Report of the Census Commissioners gives the population for those counties; but on reference to the Reports of the Registrar-General, it is found that the deaths are given for quite a different arrangement of districts. Again, if it be required to compare one district of the Registrar-General with another, the same kind of difficulty arises; for, on turning to the Census Report, those districts are in no way recognized. Precisely the same want in unity of plan is to be regretted in respect to the town districts of England, the districts of Census Commissioners constantly differing from those adopted by the Registrar-General."

Were these difficulties overcome—and no expense ought to be spared by Government to bring these two orders of reports into strict correspondence—another inquiry would still remain to be made before we could determine the true influence of locality on health and the duration of life, namely, an inquiry into the influence of employment upon health.

Not that this subject has been altogether overlooked, as the works of Thackray, Dr. Holland of Sheffield, and the recent essays of Dr. Guy, in the Statistical Journal, to say nothing of the older writers, sufficiently testify; but in some, at least, of these inquiries the want of the important element of the ages of the living, following the several occupations, has been felt and acknowledged, and till that want is supplied, we are not in a position to speak very decidedly as to the influence of employment upon health. We are not, therefore, disposed to gainsay our author's position, that "at present it is right to assume, that either employment or occupation, condition in life, or rank in society, poverty or riches, has as direct an influence on the duration of life, as peculiarity of locality or habitation; for the effect of neither one nor the other of the presumed influencing causes has yet been correctly defined;" nor shall we withhold our assent to the proposition that "in order to determine the simple influence of locality, like classes in the respective districts must be compared." This is true philosophy, and the only proceeding whereby observation can attain to the dignity and certainty of experiment. Let us now see how Mr. Neison, applies these principles to practice. He first provides himself with materials for his inquiry from two sources, namely, the quinquennial returns for 1836-1840, made under the Friendly Societies Act, and returns from Scotch friendly societies obtained by offering prizes for the best reports. From these two sources the two essential elements of all correct reasoning and calculation as to the duration of life, the ages of the living, and the ages of the dying, were obtained. There can be no doubt, therefore, of the correctness and completeness of our author's data.

A simple and natural arrangement of these facts in three groups, the first comprising benefit societies belonging to the rural districts, the second societies belonging to town districts, and the third societies established in large towns or cities, was then made, and the results for the several employments were calculated separately. By throwing the results for any single employment in rural, town, and city districts together, the influence of that employment upon health and life would be readily ascertained by comparing it with other employments, or with some common standard. On the other hand, by placing the results of the several employments under the distinct heads of rural districts, town districts, and city districts, the influence of locality is as readily determined. It is obvious that we are here provided with accurate and sufficient data for answering the question, what is the influence of employment and locality respectively upon health?

The first series of tables which we shall notice, presents in distinct columns the ages, the number living at each age, the number dying at the same age, the mortality per cent. at that age, and the specific intensity of life, (the number living divided by the mortality per cent.,) for rural, town, and city districts, separately, and for the three combined. Now as these tables present the expectation of life of that prudent part of the labouring population which possesses the means of joining benefit societies, we may expect some interesting and instructive results from conparing this important class of the community with the whole of England and Wales.

The relative value of life in this class under different circumstances of locality will be made to appear by the following statement. If we take 100,000 persons at 10 years of age, in England and Wales, and from among the members of benefit societies in rural, town, and city districts respectively, we find that while in England and Wales, half that number have died between 62 and 63 years of age, the equation takes place in rural districts, between 68 and 69, in town districts, between 64 and 65, and in city districts between 61 and 62. The rural districts, therefore, when compared with the kingdom at large, enjoy an advantage of 6 years, the town districts an advantage of 2 years, while the city districts lose one year of life. The difference in favour of the rural when compared with city districts, is no less than 7 years. Again, if we compare the specific intensity of the four classes, the vitality, so to speak, of all England, at 30 years of age, is equal to the vitality of the rural members of benefit societies at 47, of the members of town societies at 41, and of those of the city district at 33. After 50 years of age, however, the kingdom at large has a slight advantage over the towns, though it still yields to the rural districts. So also, with the expectation of life; it is uniformly better for the rural districts than for England and Wales, uniformly worse for city districts, and worse for town districts at 40 years and upwards. When the three districts are thrown together, the total results are also found to be in favour of the members of benefit societies, and they fully justify Mr. Neison's observation, that though "the circumstances in which the humble and working population of the country is placed, have generally been thought adverse to a prolonged duration of life, the healthiest life tables hitherto formed, have not shown anything so favorable as the present results, even among what are generally considered the select classes of society."

In commenting upon this remarkable fact, Mr. Neison first betrays the tendency to exaggeration which we have alluded to as one of the defects attaching to his valuable labours. The persons composing friendly societies, he observes, "are almost exclusively the hard-working members of the community, chiefly occupied in the drudgeries and toils of the mechanic arts, and consequently exposed to the inclemencies of seasons, excesses of temperature, impure atmospheres, constrained postures, and other conditions usually thought objectionable. Their incomes are very limited, affording but the scantiest and simplest means of support. Their habitations are of an inferior order, being of the cheapest kind, and consequently in the worst streets." But they are nevertheless raised above the very lowest class, for the very fact of their belonging to friendly societies, is an indication of superior industry and frugality, qualities by which they are very widely separated from the reckless and improvident, the victims, *par excellence,* of "poverty, distress, destitution, and disease." Mr. Neison would seem to be here speaking solely, or at least chiefly, of the inhabitants of large towns, and to forget that the classes who alone enjoy this longer duration of life, are the inhabitants of the country and the smaller towns, but chiefly the former, while the inhabitants of the larger towns, members of benefit societies though they be, fall far short in this respect of the average of all England. Here, then, we think that Mr. Neison's strong views have led him to exaggerate the bearing of his facts on the sanitary question.

We nevertheless fully agree with Mr. Neison, in thinking the superior value of life among the members of friendly societies, a very remarkable and important feature in the inquiry, in which we willingly follow him, "From what source or class does the excess of mortality, which makes up the general average of the community, arise?" We learn, in the first place, that the upper classes of society are among the contributors to this excessive mortality. This statement, though at variance with the prevailing opinion, is fully borne out by facts and arguments adduced by Mr. Neison, and by tables which will be found at pages 36 and 37 of his work. The first table places side by side, the expectation of life among the families of the peerage and baronetage, as calculated by Mr. Neison from facts collected by Dr. Guy, and the expectation of life among the members of benefit societies in the most unhealthy city in England—Liverpool. The result is very remarkable. The expectation of life among the aristocracy exceeds that of the numbers of friendly societies in the city of Liverpool, by somewhat less than a year, throughout the whole term of life, and falls short by two years or more of the expectation for the members of friendly societies in city districts. *A fortiori,* therefore, it is lower among the aristocracy than for the male population of England and Wales. In further confirmation of the statement that the higher classes have a shorter expectation of life than the average of the entire community, a second table is given contrasting the expectation of the members of friendly societies, belonging to 16 trades, in the rural districts with the aggregate observations of assurance companies, and the tables calculated by Mr. Finlaison, on the lives of the nominees of the Government tontines and annuities. Now, here we have selected lives from among the higher and middle classes, contrasted with selected lives among the labouring population, and yet a result favorable to the latter class. To prove this it will suffice to compare the three classes at the same age. If we take, for instance, 20 years, the vitality of the three classes will be represented by the following numbers.—Friendly societies 44, assured lives 40, government annuitants $37\frac{1}{2}$. At the age of 30, the numbers are $37\frac{1}{4}$, $33\frac{1}{4}$, and $32\frac{1}{2}$; at 40—30, 26, 26; at 50—23, $19\frac{1}{2}$, $19\frac{1}{2}$, and at 60—$16\frac{1}{4}$, $13\frac{1}{2}$, $13\frac{1}{2}$.

We cannot, therefore, withhold our assent from the proposition that the labouring classes have a better expectation of life than the higher orders, and that the lower duration of life among the latter is one of the sources of the excess of mortality which makes up the general low average of the community as compared with the members of benefit societies.

Nor are we disposed to differ from the sentiments conveyed in the following passage: "The blessing thus bestowed on the frugal and industrious workmen of the country composing friendly societies, in having granted them, as appears by the present inquiry, a prolonged duration of life, must, therefore, be recorded as a really true and distinctive feature of that class of persons, and is, no doubt, the result of their simple and uniform habits of life, and the more regular and natural physical exercises to which they are habituated." We think it highly probable, moreover, that "it could be clearly shown, by tracing the various classes of society, in which there exists sufficient means of subsistence, beginning with the most humble, and passing into the middle and upper classes, that a general deterioration in the duration of life takes place; and that just as life, with all its wealth, pomp, and magnificence, would seem to become more valuable and tempting, so are its opportunities and chances of enjoyment lessened. As far as the result of figures admit of judging, this condition would seem to flow directly from the luxuriant and pampered style of living among the wealthier classes, whose artificial habits interfere with the nature and degree of those physical exercises which, in a simpler class of society are accompanied with a long life."

This view is strengthened by a comparison instituted in Dr. Guy's essay already referred to, between the members of the families of the peerage and baronetage, and the successors to titles among the peerage. At 25 years of age the expectation among the former class exceeds that among the latter by upwards of 5 years, and by upwards of 2 years at 35 and 50 years of age. "This difference," as Dr. Guy observes, "can scarcely be due to the mode of calculation, and therefore gives rise to a question of some interest. The families of the peerage and baronetage comprise a large proportion of persons urged by ordinary motives to wholesome exertion of body and mind, while the expectants of title may be fairly presumed to have a greater command of the means of self-indulgence, and less motive to those efforts, whether mental or bodily, by which men may be said to earn health and long life. It is not a little remarkable that the expectation of life among the male members of the families of the peerage and baronetage should exceed by from two to four (five?) years the expectation of life for the same age among the successors to titles; and that the expectation of life in the former class falls short in a similar manner, and to a similar extent, of that of the entire kingdom and of the lives insured in the principal insurance offices. There is here a coincidence which cannot be overlooked, and a fair ground for answering the question already proposed in the affirmative. In the unlimited command of the means of dangerous self-indulgence, and in the absence of the common motives to wholesome exertion, the expectants of titles differ as much from the other members of noble families as these differ from the mass of mankind; and the effect in each case displays itself in broken health and a shorter average duration of life. When the duration of life shall be accurately ascertained for all the several classes of society, it will probably be found that the labouring man, placed above want, but always dependent upon his own exertions, attains a higher average age, as he undoubtedly reaches a higher extreme, than his richer and more luxurious superior."

From all that we have stated it must be quite obvious that the classes which by their high rate of mortality lower the duration of life of the whole community are the higher and middle classes and the inhabitants of large towns, and that the laborious inhabitants of the rural districts and smaller towns tend to increase that duration. That the labouring classes of large towns generally have a low average duration of life may be inferred from the fact that even members of benefit societies inhabiting these towns enjoy a less favorable term of life than the average of all England. Mr. Neison seems to have overlooked this fact so clearly established by his own tables, when he represents the mortality of England as being constituted by that of the members of friendly societies added to that of the middle and upper classes, leaving as the chief cause of the low average duration of the entire community the high rate of mortality prevailing among "the improvident and reckless, the poor and the destitute, who are exposed to the inclemencies of the seasons, the fluctuations of trade, and fall victims to epidemical and other diseases."

It may be held to be perfectly certain, and it is a fact more and more confirmed by every fresh inquiry, that there is an excessive mortality and low duration of life in the inhabitants of large cities, and that not during infancy alone, but at every period of life. With the mortality during infancy we have here no concern, further than to signalize it as a proof that towns are in themselves injurious to health and life; but if we confine our attention to the case of adults it is clear that a high mortality may be due to two causes—the inherent unhealthiness of a town life, or the concentration in towns of employments in themselves unwholesome. This is the next question to which Mr. Neison addresses himself, and with a very marked leaning towards the latter alternative. We will follow him step by step in his inquiry.

The first result at which he arrives is that the agricultural labourers are longer-lived than the total of the inhabitants of rural districts; from which it may be reasonably inferred that the luxury and self-indulgence of the better classes, and the more confined and sedentary life of the tradesmen and artisans inhabiting those districts is, in a very marked degree, unfavorable to health and longevity. The difference in favour of the labourer, at the earlier periods of life, is about two years. The position of the labourer appears still more favorable, when we contrast his expectation of life, not with the total including his own class, but with the residue. Thus, while half the population of labourers dies off in the 72d year, and half the general average in the 69th year, half the residue have perished in the 65th year. The labourer, therefore, enjoys an advantage over the residue of about seven years; a difference which Mr. Neison attributes solely to difference of occupation, to the exclusion of the "*supposed* contaminating influences of ill-ventilated houses, narrow streets, bad sewerage, poisoned air, epidemic town fevers, and factory restraints." We have italicised the first word of this quotation to attract attention to this fresh proof of our author's scepticism, which, as we think, is scarcely justified by the facts of the case. Though it is quite true that our rural villages in which the residue, with the exception of country gentlemen and farmers, reside are not cursed with narrow streets or factory restraints, they are not quite so free as Mr. Neison seems to assume from ill-ventilated houses, bad sewerage, poisoned air, and epidemic fevers. On the contrary, ventilation is often quite as much neglected in villages as in large towns; sewerage is not bad simply because it does not exist at all; the air is not entirely free from unwholesome exhalations, nor is epidemic fever quite so rare a phenomenon as the above quotation would seem to assume. To all these influences, in a degree of intensity not much inferior to that which prevails in large towns, the inhabitants of rural districts are exposed, and those most who are most within doors, and most debarred from exercise. The error into which, as we believe, Mr. Neison has fallen is the not uncommon one of regarding occupations only in one point of view, namely, as entailing more or less exercise, or demanding a more or less constrained and irksome posture of body, without considering all the circumstances by which they are distinguished, and keeping out of sight the vitiated atmosphere in which all in-door employments are carried on. The agricultural labourers combine in their greatest possible degree, exercise and pure air, while those who follow other employments have too little of the one or the other, or of both: the former are less exposed to the causes of disease, and better prepared by healthful exercise to resist them, the latter are less favorably circumstanced in both these respects. This, then, is the secret of the higher expectation of life enjoyed by the agricultural labourer.

It will be seen that we differ from Mr. Neison, not in denying that employment has a very marked effect on the duration of life, but in asserting that one employment is more wholesome than another, because it exposes those who follow it in a less degree to the causes of disease which lurk in and about all the habitations of men, whether in villages, towns, or cities.

As to the necessity of taking into account the nature of the employments followed by the inhabitants of civic and rural districts, and of comparing men following the same occupation in the two localities, in order that we may ascertain the real influence on adult life of town and country respectively, we are in perfect agreement with our author. The necessity of such a mode of proceeding is too obvious to be denied, and until such a comparison is instituted we are bound to suspend our judgment as to the precise influence of locality on the health of our adult population. Mr. Neison has the materials for this exact comparison in his own hands, and we trust that he will not lose the opportunity of solving so interesting and important a problem. In the meantime, we are willing to hazard the conjecture, that the comparison will confirm the views of those who attribute the unhealthiness of our large towns to defective structural arrangements and overcrowding of dwellings and places of work, while it will somewhat disappoint those who have shown a tendency to an exaggeration of the opposite kind to that which Mr. Neison seems to us to have fallen into. But whatever may be the result of such a comparison, the high mortality of children in large towns is a fact which no argument drawn from the prevalence of unwholesome employments upon adults can shake,—a fact which proves beyond doubt or cavil, that cities are in themselves injurious to health and fatal to life.

But though we differ from Mr. Neison in the interpretation of facts we willingly own the great obligations under which he has laid us for the facts themselves, and we proceed to select from his ample stores some of the more striking and important of them.

Having shown the difference existing in the duration of life of the agricultural labourer and of the remnant of the inhabitants of rural districts, and thus proved the marked influence of employment upon health, where that influence would least have been sought for, he proceeds to compare the rural, town, and city districts in the aggregate with the special employments of the miner, the baker, the painter, and the clerk. Taking the age of 30 years, it appears that while the aggregate at that age enjoys an average expectation of nearly 37 years, the miner has about 33, the baker 32, the painter 30½, and the clerk 27½.

"It will no doubt cause some uneasiness in the minds of inquirers to find, that so highly important and industrious a class of men as clerks should stand lowest in the scale of the above employments, and that from 20 to 60 their expectation of life should be only 75 per cent. of the general average. The expectation of life among plumbers, painters, and glaziers in the same period is equal to 81 per cent., miners 85 per cent., and bakers 88 per cent. of the general average." It is doubtless a source of uneasiness, and one which must increase with civilization, but it cannot surprise us for a moment when we consider the sedentary life, the long hours, and the sort of air in which these unfortunate persons have to pursue their occupations. The fate of the tailor, the compositor, and the drapers' assistant is as much, if not more, deplorable, and we are sorry that Mr. Neison has not extended his comparison to these unhappy victims of overwork in a close, heated, and impure atmosphere.

We are next presented with tables contrasting the expectation of life of the aggregate of city districts with Liverpool, and the members of friendly societies with the entire population; a contrast which confirms the results of recent inquiries, at the same time that it proves the better sanitary state of the members of benefit societies. Our author shall explain in his own way the excessive unhealthiness of Liverpool. He says:

"A careful consideration of all the preceding observations, it is believed, will be sufficient to show that the excessive mortality of the general population of Liverpool must be due to some other cause than simply that of locality. The persons over whom the observations in the first column extend, being members of friendly societies, and almost exclusively workmen and mechanics, of necessity inhabit the inferior class of houses, in the worst conditioned streets; and it is therefore impossible that they can escape the contagious effects of the pestilential diseases supposed to be the scourge of unhealthy neighbourhoods; *and, admitting this*, the results given for the friendly societies must evidence all the legitimate effects due to locality; and therefore the excessive mortality of the general population is due to some other cause—such as the poverty and distress, which, unhappily, are allowed to remain too much neglected in the large manufacturing and commercial towns of the kingdom."

The italics in this passage are our own, and by adopting them we wish to intimate that we do not admit the position, at least to the extent to which it is pushed. We think it highly improbable that men who have the means or the prudence to belong to friendly societies form any considerable proportion of the dwellers in the cellars or narrow courts of Liverpool. And, be it recollected, the unhealthiness of Liverpool is not attributed by those who know anything at all of the matter to its locality, but to the peculiarly bad structural arrangements with which it abounds. Liverpool is pre-eminently a city of cellars and courts; and it is these cellars and courts, and not the locality of the city, nor the peculiar destitution of its inhabitants, which must bear the blame of its excessive mortality. Like all other large cities it abounds in unhealthy trades, and has its share of the poor and destitute, but there is no ground for supposing that in the aggregate its trades are more unhealthy, or its poor more numerous than those of other large cities which present a far more favorable figure of mortality. Are the employments of its inhabitants, taken one with another, more unhealthy than those of Birmingham or Sheffield? We think not; nor do we imagine that its poor are more numerous. Our author insists strongly upon the necessity of taking into account the occupations of the inhabitants of the cities which we make the subject of comparison. We cordially agree with him; but we submit that we ought to be equally careful in contrasting their places of abode. If it would lead to serious error to compare the rural labourer with the clerk, it would be to the full as fatal to accuracy to institute a comparison between the dweller in an open street and the miserable occupant of a cellar. If the comparison between Liverpool and Birmingham would be inexact because the one abounds in dock labourers and the other in mechanics, surely it would be equally inconclusive, as far as the influence of mere locality is concerned, seeing that in the one there are several thousand of cellar-dwellings, while in the other such disgraceful habitations are altogether unknown. The precise influence of locality upon health, therefore, is a problem of very difficult solution, for it is not likely that we shall be able to find a group of conditions—employment, place of residence, wages, habits of life, &c.—precisely, or even very nearly, the same in any two cities under the sun. We must be content, therefore, with approximations and probabilities, receiving with due acknowledgment such valuable suggestions as those which Mr. Neison has thrown out, but not allowing ourselves to be driven from a position which we have taken up and fortified by fact and argument, till we are either fairly beaten, or see that our adversary is prepared to overwhelm us with weightier metal than our own. At present we are determined to hold out, and fight the battle of locality, or to speak more correctly, of habitation *versus* employment.

We are obliged to dismiss an interesting chapter on the duration of life in Scotland, with the brief remark that in that country there is one city which presents a worse sanitary state than unhealthy Liverpool itself, we mean Glasgow. At 30 years of age, the expectation of life for Liverpool is 27 years, for Glasgow less than 25; and a similar difference exists at other ages. Surely this is a strong argument in favour of the extension of the provisions of the sanitary bill recently laid on the table of the House of Commons to Scotland.

But what of unhappy Ireland? Mr. Neison's inquiries do not extend to her; we must, therefore, turn for a moment to the work of Mr. Willis, where we find (at p. 18,) a life-table founded on facts carefully collected by inquiries among the labouring population of Dublin. Though this table does not admit of exact comparison with those of Mr. Neison, inasmuch as the life-tables for Liverpool and Glasgow, are formed for the whole population of those cities, yet it may be interesting to our readers to know how the results for this one class stand as compared with those obtained for the unhealthiest cities of England and Scotland. If we take as before the age of 30, the expectation of the town of Liverpool being 27 years, and for Glasgow 25 years, that for the labouring class of Dublin is 24 years. For more advanced periods of life, however, the Dublin table presents more favorable results. Thus at 50 years, the expectation for Liverpool and Dublin being 16, that for Glasgow is $14\frac{1}{2}$ years.

The greater part of the inquiries on which the life-table is founded, were made in the crowded parish of St. Michan, each house in which parish, contains on an average $16\frac{1}{4}$ persons, (St. Giles' in London, can only boast of 11 to a house.) Of this district, Mr. Willis gives a description which we cannot forbear from quoting, so strongly does it remind us of what we have recently read of the state of some parts of the unhappy country of which this is the capital.

"There are no gentry within the district, and the few professional men or mercantile traders, whom interest may still compel to keep their offices here, have their residences in some more favoured localities. This parish, that within the last thirty years might boast of as large a proportion of professional and mercantile wealth as any in the metropolis, is now the refuge of reduced persons from other districts; and very many of the houses then occupied by respectable traders, are now in the possession of a class of men called 'house-jobbers,' who re-let them to poor tenants. These jobbers have no interest in the houses save their weekly rent; the houses, therefore, undergo no repair; the staircases, passages, &c., are all in a state of filth; the yards in the rear are so many depots of putrid animal and vegetable matter; and if a necessary be in any of them, it frequently is a source of further nuisance. The courts and back places are, if possible, still worse, and are quite unfit for the residence of human beings. They are almost all closed up at each end, and communicating with the street by a long narrow passage, usually the hall of the first house, and not more than three or three and a half feet wide. Pipe-water, lime-washing, dust-bin, privy—these are things almost unknown. The stench and disgusting filth of these places are inconceivable, unless to those whose harrowing duty obliges them to witness such scenes of wretchedness. In some rooms in these situations it is not an infrequent occurrence to see above a dozen human beings crowded into a space not fifteen feet square. Within this space the food of these wretched beings, such as it is, must be prepared; within this space they must eat and drink; men, women, and children must strip, dress, sleep. In cases of illness the calls of nature must be relieved; and when death releases one of the inmates, the corpse must of necessity remain for days within the room. Let it not be supposed that I have selected some solitary spot for this description: no, I am speaking of an entire district, and state facts incontrovertible. I indulge in no theories as to the causes which produce this state of things, but I may state the results. They are—that every cause that can contribute to generate contagion exists here in full vigour, and that disease, in every aggravated form, with all its train of desolating misery, is rarely absent."

After this description we shall scarcely be surprised to hear that "eighty cases of fever, including relapses, were said to have occurred in one house in the course of twelve months." "Fifty persons have been admitted to hospital from another within a year." "Thirty patients from another within eight months." "Nineteen from a fourth in six weeks." "The inmates of a house which was thrice lime-washed in the space of a few weeks, were as often readmitted to hospital, in consequence of sleeping in their infected bedding."

Poor Ireland! Turn where we may, you present us with the same sad picture of destitution and misery. The records of the land-commission, the letters of the "Times commissioner," the census, and the writings of those who treat of sanitary matters, all tell one consistent tale. Poverty, and his haggard offspring, misery, disease, and crime, possess the land, and all the efforts of all the governments in the world cannot remove them. We must be satisfied with knowing that it is so, and go on learning till knowledge bears its certain fruit. To those who seek for knowledge of this sort, we recommend Mr. Willis's work, and resume our notice of Mr. Neison's labours.

The first part of his work treated of the influence of locality, and incidentally of employment, on the duration of life; the last two chapters treat of the influence of locality on sickness, with an application of the results to the purposes of friendly societies. Our readers will soon perceive that this is a most important portion of the work before us. We shall treat this part of the work with all the respect which we have already paid to the first part, and shall analyse the results with the same minuteness.

We are first presented with a table showing the average sickness per annum, in weeks and fractions of a week, in the rural, town, and city districts, from which it appears that sickness, speaking generally, is at a minimum in rural districts, more rife in town districts, and still more prevalent in city districts, thus showing a general agreement with the mortality. The correspondence, however, is only general; for tables subsequently adduced, show that the relation of cause and effect generally supposed to exist between sickness and mortality does not always find place, but that, on the contrary, "the highest ratio of sickness is sometimes found associated with a favorable rate of mortality." "For example, labourers, although influenced by the most favorable rate of mortality, are found to be subject to as high an amount of sickness, as the general average." "Bakers, also, at the early and middle periods of life, are less subject to sickness than the general average, and among them there is likewise a higher mortality. The class butchers seem to experience a very high rate of mortality, although not subject to above the average amount of sickness." Tailors and clerks, again, are subject to a very high rate of mortality, without being subject to so much as the average of sickness.

It must be recollected that the sickness here spoken of is benefit society sickness, or, in other words, disabling sickness, and "that the same trivial circumstances which would be sufficient to disable sawyers, colliers, and miners, would have little effect on the tailor or the clerk." Sawyers, colliers, and miners are also subject to accidents and various injuries, which cannot be considered constitutional disease or sickness; yet it entitles them to relief from benefit societies, and they will of course be returned on the sick list. Tailors and clerks are less subject to these accidents, and accordingly their sickness is also less." When these circumstances are taken into the account, it must be evident that the true relation of sickness and mortality is not set forth in the returns of benefit societies. At the same time it must be admitted to be extremely probable that in some employments there is a great liability to slight attacks of disease, in others, a liability to one or two dangerous and fatal maladies. But we must pass on from this speculative point to one of the highest practical importance; one in which the well-being of thousands of our most deserving fellowcitizens is involved; the observed amount of sickness, as the basis of the calculations and practical operations of benefit societies. And here we must present our readers with a short table contrasting the results obtained by the Highland Society and those of Mr. Ansell with the more recent calculations of Mr. Neison.

*Annual Sickness to each person in weeks:*

| Age. | Highland Society. | Ansell. | Neison |
|---|---|---|---|
| 20 | ·575 | ·776 | 840 |
| 30 | ·621 | ·861 | ·911 |
| 40 | ·758 | 1·111 | 1·559 |
| 50 | 1·361 | 1·701 | 1·960 |
| 60 | 2·346 | 3·292 | 4·166 |
| 70 | 10·701 | 11·793 | 14·039 |

On this table the author has the following remarks.

"The remarkable increase in the amount of sickness, as shown by the present results, beyond the two other tables, will no doubt appear very startling to those not intimately familiar with the condition of friendly societies throughout the country. The rate of sickness as given in the table of the Highland Society, has been long and generally acknowledged to be much below the actual average, and even so far back as 1825 it was thought unfavorably of by a Committee of the House of Commons. It is unnecessary to enter into the objections against the nature and source from which the data for the Highland Society's tables were obtained, as that subject has been amply discussed elsewhere. For some time after Mr. Ansell's work appeared, it was thought that contributions calculated according to the increased amount of sickness shown in his tables would render friendly societies perfectly safe; but instances occur almost daily of societies breaking down whose contributions approximate to these tables; and recently the increased amount of sickness has become so apparent to the members of some of the best regulated societies, that meetings have been held, and reports of a very clear and opposite kind published, pointing to the increased amount of sickness as the cause of their falling condition. A knowledge of circumstances of this kind first led to the present inquiry, the original object of which was simply to answer the question —whether friendly societies were subject to a higher rate of sickness."

We shall not follow Mr. Neison into his discussion of the causes of the difference existing between results so recent as those of Mr. Ansell, and his own; but content ourselves with expressing our entire confidence in his own tables, and the strong sense we entertain of the benefit which he must be the means of conferring on the societies in whose welfare he has taken so deep an interest. We can scarcely conceive a more important subject than that to which he has directed our attention. If it be important to encourage to the utmost habits of forethought and frugality it is little short of a crime to disappoint the cherished hopes of the deserving persons who display these virtues by negligently leaving the solution of the question on which a future provision must rest to the chance efforts of individuals. We, therefore think that there is much force and justice in the following observations:

"To those interested in the progress of friendly societies, the results are highly important, as they will demonstrate the impossibility of permanence in those institutions on their present foundation. Considering the immense number of those societies which have broken down, it is lamentable to think that so little should have been done to ascertain the real nature and extent of the risks to which they are subject. It is still more remarkable that so many legislative enactments should have occupied the attention of the government of the country from time to time, and that committees also of the House of Commons should have had the condition of those societies for several years under consideration, without any practical measure being carried out for collecting and arranging data in a proper shape to point out the true character of the liabilities to which they are subject. In fact, the stimulus given to the formation of those societies by some recent acts of Parliament should be regarded as an evil rather than as a benefit to the working classes. An immense number of societies were formed in a very short period, and their contributions regulated by the most delusive and inadequate data, so that at the present time very few are to be found calculated to survive many years. Under a scientific and amply-developed system, those societies would be calculated, in a few years, to completely remove the cause of nearly all that poverty, distress, and misery, which haunt our manufacturing towns, and fill our workhouses with the working classes of the country; but owing to the imperfect and unstable foundation on which they are at present built, instead of being a help and support to a poor man, they involve him in those difficulties for which he might otherwise have provided. On becoming a member of such a society, he reasonably looks forward to it as a support for his declining years, and a protection during periods of sickness and disease; but, ultimately, at the very time when assistance is required, he discovers that the society has been formed on a ruinous plan, that the increasing years and infirmities of its members have absorbed all its funds, and that those surviving must be thrown destitute on the parish or a public charity. It is thus, by the most ill-conceived of all proceedings, the legislation of the government has hitherto tended. Every facility and encouragement are given to the formation of societies, without any help or information for their management or guidance. The ship is cast upon the waves without a rudder or a compass, and the safety of the vessel left to accident or chance."

There is a glimpse here of what will assuredly some day come to pass, when the government shall be alive to the value of the principle of insurance carried out to its full extent, and substituted for the clumsy, cumbrous, and costly machinery of poor-laws and union-houses, and all the wasteful extravagance of our present system of mending, and soldering, and patching; when in all things the great practical motto, "*Prevention better than Cure,*" shall become the motto of the state, and the system of *Laissez faire,* with all its fearful offspring of misery, disease, and crime, shall cease and determine. Can any one doubt for an instant what the effect would be of a comprehensive system of benefit-societies, coextensive with savings'-banks, guaranteed by government against the accident of limited observation or incorrect calculations, and in which the labouring classes might place implicit reliance? We may rest assured that, ere many years had passed over our heads, some of our unions would be found too large, and our prisons perchance to offer too much accommodation. The principle of assurance, like our civilization, is but in its infancy, and the art of government in its cradle. But the nineteenth century has something better than railroads in store for us, or we greatly mistake the signs of the times.

We are warned by the length to which the foregoing remarks on this truly valuable work have extended, that we must hasten to a close; but we cannot conclude without quoting a somewhat lengthened passage, which embodies our author's views on one of the greatest practical questions of the day—the sanitary question:

"If in any public inquiry it should be attempted to ascribe the increased amount of sickness in the town districts to the less healthy nature of the districts, or their peculiar local influence on health, the conclusion would certainly be fallacious. Precisely similar arguments to those made use of in reference to the mortality of those districts, will explain the differences in the ratio of sickness in the same places; and it is therefore to be inferred, that whatever sanitary regulations may be carried out for promoting the health of towns, the wide distinction between the rates of sickness and mortality in particular districts will still not disappear. The cause of that difference is beyond the reach of any sanitary measure; and unless a change were to take place in the character and machinery of the manufactures of a town, by which the workmen would be habituated to less restrained but more active and complete physical exercises, no improvement in the state of health is to be looked for. The evils, so far as relates to health, represented to exist by some writers to so frightful an extent, and to connect themselves with inferior sewerage, filthy streets, and ill-planned houses, are certainly overstated by them. The data brought forward have generally been of the most indefinite and insufficient nature; and when, in connexion with this, the erroneous methods employed, and the promiscuous manner in which their figures are generally combined, are kept in view, it must seem surprising that the thinking and intelligent portion of the community should have given their opinions any credence, or believed their conclusions entitled to so much weight. Perhaps no statistical facts are better established than the duration of life among the middle and upper classes of this country; and if the data brought forward in this paper be received as of sufficient merit to represent the duration of life among the working classes, it will then appear clear that any important change to be hoped for in the value of life in the town districts, must be effected through other means than sanitary regulations. Those persons purchasing government annuities, and having dealings with assurance companies, are certainly beyond the reach of any improvements to be introduced by local regulations; and if cleanliness of habit, comfort of dwellings, and fresh air be of themselves powerful elements in raising the standard of life, their influence should be felt among that class of persons. But what are the actual results? The poor workmen inhabiting the miserable streets of our large towns, and inhaling their supposed noxious vapours, are actually longer lived than the affluent and upper classes, whose easy circumstances enable them to inhabit comparatively the palaces of the kingdom. It is evident, from the disparity in the value of life among different classes of workmen, whose conditions as to whatever is within the scope of public sanitary measures are the same, that other elements must exist having a powerful influence on the duration of life. It would further appear, by viewing the various classes of society more in connexion with the physical exercises to which they are habituated, than in connexion with their moral position and rank in society, and consequently with their sanitary condition, that a better clue will be found to the differences in the duration of life. It is not to be expected that any arrangements whatever as to the drainage and planning of streets, are likely to add to the longevity of a tailor; but if it were possible to give his frame the physical exercises of a ploughman, twenty per cent. would be added to the duration of his life. Neither is it to be thought that the plumber, painter, and glazier is to be relieved from the poison of the metallic emanations to which he is subject; nor that the clerk can inhale the fresh air and indulge in those exercises necessary to develop his physical constitution, while he follows the drudgeries of the counting-house. It is an aggregation of these, and other employments similarly conditioned, which make up the excessive mortality of our large towns; and since it has been shown in the preceding pages, that this class of lives is also less healthy even in the country districts, and that the town populations are chiefly made up of persons following such occupations, the legitimate result to be expected is a shorter duration of life in towns, independent of any local influence on health. If improvements and changes are to be effected in the sanitary regulations of our large towns and cities, let them at once be carried out—not upon the necessity of such municipal innovations to avert a pestilential havoc in human life—but on the true merits of the question—the comforts, conveniences, and elevation of taste and moral purity thence arising."

These opinions of Mr. Neison, though, as we think, hastily formed and greatly exaggerated, are entitled to be treated with the respect due even to the errors of one who spares no pains or expense in collecting facts, and who exhibits unusual assiduity in using them. But we cannot withhold the expression of our surprise and regret that Mr. Neison should have treated with so little respect the conclusions of the many men of science, who have concurred in tracing the high mortality of our large towns to the causes to which he attaches so little importance. It is at least within the bounds of possibility that similar fallacies to those which Mr. Neison assumes to have misled Mr. Chadwick, Dr. Southwood Smith, the members and witnesses of the different health-commissions, and the government itself, may have crept in among his own conclusions.

The higher classes may be short-lived in spite of their broader streets, and larger houses, and better food, and warmer clothing, and greater personal cleanliness ; just as the lower classes may be short-lived from the want of all these advantages. Luxury and self-indulgence may be to the full as fatal as hardship and privation. A king may not be one whit healthier or longer-lived than a beggar, a peer than a tailor, a member of the House of Commons than a delegate of a trade's union, an author than a compositor ; but *non constat* that the unhealthiness of the extremes of the social scale may not spring from such different causes as to render any comparison between them ridiculous. The one may take as much poison into his stomach as the other into his lungs, the chief difference between them being, that the rich man has more control over his cook than the poor man has over his employer. It will be much easier for the one to order a plain dinner, than for the other to procure a single breath of fresh air. When Mr. Neison shall have succeeded in explaining away the eighty cases of fever, including relapses, occurring in one house in Dublin in the course of twelve months ; the fifty cases occurring in another in the same period ; the thirty patients in eight months from a third ; and the nineteen in six weeks from a fourth, and scores of similar facts occurring in all the large towns of the empire, without the aid of defective drainage and sewerage,—we will discard this item of our sanitary creed. When he shall have instituted an exact comparison between the tailor, compositor, or needlewoman, working in a crowded, heated, and unventilated apartment, and the same class following the same avocations for the same number of hours a day, in a cool and pure atmosphere, and shall succeed in proving that the one class is to the full as healthy as the other, we will give up all faith in ventilation, and embrace his own theory of deficient exercise. All this, and something more, must come to pass before our friends of the Health-Commission and their allies can be forced from the strong position which they have taken up. They may have fortified themselves with some worthless arguments, and entrenched themselves behind some showy fallacies, but in the main we are convinced that they will stand their ground, till they find Mr. Neison himself of their mode of thinking. Not content with advocating "municipal innovations," as conducing to "the comforts, conveniences, and elevation of taste and moral purity, thence arising," they will continue to urge them with all their power and influence as essential to the enjoyment of such health as is compatible with the disadvantages to which the social occupations of the labouring class expose them. In the mean time, they cannot be insensible to the great value of Mr. Neison's labours, and they hail him as a fellow-worker with themselves in that stubborn and tangled field of mingled facts and fallacies, where the wheat and the tares still grow together, and the weeds cling even to the sheaves.

---

ART. II.

